# After the Fall: Gastrointestinal Illness following Downpours

**DOI:** 10.1289/ehp.123-A243

**Published:** 2015-09-01

**Authors:** Carol Potera

**Affiliations:** Carol Potera, based in Montana, also writes for *Microbe*, *Genetic Engineering New*s, and the *American Journal of Nursing*.

Many older cities have “combined” sewer systems that collect both sewage and stormwater runoff in the same pipe for transport to a wastewater treatment plant. During heavy rainfalls, these combined systems can become overloaded, and water utilities must discharge the excess—what’s known as a combined sewer overflow (CSO)—into rivers and other water bodies, which may be the local source of drinking water. Bacteria, viruses, and protozoa in these untreated releases can cause waterborne diseases.^1^ In this issue of *EHP*, researchers report that emergency room visits for gastrointestinal (GI) illnesses increased after heavy rainfalls in areas of Massachusetts served by combined sewer systems, offering evidence that CSOs may adversely affect human health.^2^

Swedish researchers had reported earlier that GI-related calls to nurse advice lines in Gothenburg, Sweden, increased by up to 30% in the 5–6 days after heavy rainfalls.^3^ The Swedish team suspected that pathogens in drinking water caused at least some of the callers’ GI symptoms. They did not track water contaminants themselves but suggested CSOs could be related.

**Figure:**
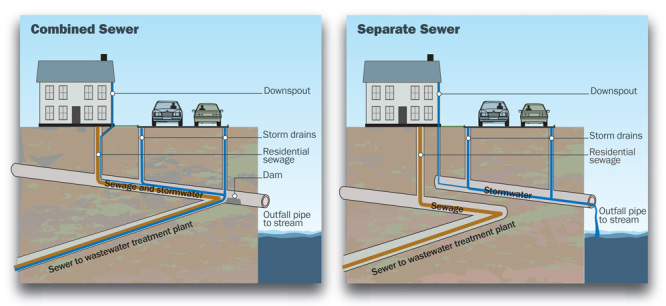
Combined sewer systems use the same pipe to route stormwater and sewage to a wastewater treatment plant. These systems are designed to discharge excess flow into a nearby water body. Modern separate sewer systems route sewage to a treatment plant via one pipe while another directs untreated stormwater into water bodies. EHP

For the current study, investigators utilized data on daily visits to emergency rooms for GI illnesses after extreme rainfall events in Massachusetts from 2003 to 2007. They assessed three study areas. The first included 11 towns with combined sewer systems that discharged into the Merrimack River, a source of drinking water for all 11 towns. The second included 24 towns with combined sewer systems whose overflow ran into Boston Harbor, which is used largely for recreation. The third included 9 towns with modern “separate” sewer systems that transported sewage independently of storm runoff.

During the study period, 18 extreme rainfalls occurred, defined as the 99th percentile or greater—this equated to 1.33, 1.60, and 1.97 inches of rain per event at the three sites, respectively. In towns where CSOs flowed into the Merrimack River emergency room visits increased by an average 13% for all ages and an average 32% for people older than 65 years about a week after extreme rainfalls. In comparison, no unusual rise in visits occurred at hospitals in towns that did not have combined sewer systems or where CSOs were discharged into recreational waters.^2^

“More heavy, sporadic rainfalls are predicted as climate changes,” says first author Jyotsna Jagai, an epidemiologist at the University of Illinois at Chicago. “Our findings suggest that drinking water quality may be adversely impacted by the presence of CSOs that discharge into drinking water sources after heavy rainfall, although we did not directly measure water quality,” Jagai says.

Combined sewer systems still serve 772 communities, primarily in the Northeast, Great Lakes, and Pacific Northwest regions.^4^ Fixing the problem requires building expensive, new infrastructure to handle sewage and runoff separately.^5^

“Many people take water and wastewater infrastructure for granted. Jagai’s study highlights the importance of investing in our aging water infrastructure, especially in the face of climate change,” says Karen Levy, an assistant professor at Emory University’s Rollins School of Public Health. Levy did not participate in the study.

Water authorities must report CSO releases to regional U.S. Environmental Protection Agency offices, which also manage the discharge permitting process. Jagai plans to collect agency data on CSO releases to further assess emergency room visits soon after reported events in Midwestern cities. This will allow the researchers to look for associations between known CSOs and GI illness, rather than use heavy rainfall as a predictor of CSO events.

Future results “could be bolstered by water quality studies to identify when water contamination hits a tipping point that may have health effects,” Levy says. “This could help to devise monitoring programs and early warning systems for actions such as advisories for the public to boil water.”
